# Early endosome motility mediates α-amylase production and cell differentiation in *Aspergillus oryzae*

**DOI:** 10.1038/s41598-017-16163-1

**Published:** 2017-11-17

**Authors:** Yusuke Togo, Yujiro Higuchi, Yoshinori Katakura, Kaoru Takegawa

**Affiliations:** Department of Bioscience and Biotechnology, Faculty of Agriculture, Kyushu University, 6-10-1 Hakozaki, Fukuoka, 812-8581 Japan

## Abstract

Recent research in filamentous fungi has revealed that the motility of an endocytic organelle early endosome (EE) has a versatile role in many physiological functions. Here, to further examine the motility of EEs in the industrially important fungus *Aspergillus oryzae*, we visualized these organelles via the Rab5 homolog AoRab5 and identified AoHok1, a putative linker protein between an EE and a motor protein. The *Aohok1* disruptant showed retarded mycelial growth and no EE motility, in addition to an apical accumulation of EEs and peroxisomes. We further demonstrated that the *Aohok1* disruptant exhibited less sensitivity to osmotic and cell wall stresses. Analyses on the protein secretory pathway in Δ*Aohok1* cells showed that, although distribution of the endoplasmic reticulum and Golgi was not affected, formation of the apical secretory vesicle cluster Spitzenkörper was impaired, probably resulting in the observed reduction of the *A. oryzae* major secretory protein α-amylase. Moreover, we revealed that the transcript level of α-amylase-encoding gene *amyB* was significantly reduced in the *Aohok1* disruptant. Furthermore, we observed perturbed conidial and sclerotial formations, indicating a defect in cell differentiation, in the *Aohok1* disruptant. Collectively, our results suggest that EE motility is crucial for α-amylase production and cell differentiation in *A. oryzae*.

## Introduction

The early endosome (EE) is an organelle in the endocytic pathway in filamentous fungi that is constantly moved along the microtubule (MT) by two motor proteins, kinesin and dynein^1^. The molecular mechanisms underlying how EEs exhibit motility have been intensely investigated in the model filamentous fungi *Ustilago maydis* and *Aspergillus nidulans* ^2,3^. The motility of filamentous fungal EEs was first visualized in *U. maydis* with Yup1, a soluble *N*-ethylmaleimide-sensitive factor attachment protein receptor (SNARE), in cells that were also stained with the endocytic marker dye FM4-64^4^. Subsequent studies have characterized the EE-specific small GTPase Rab5 in several filamentous fungi^5–7^. Rab5-positive EEs move along bipolar MT arrays: movement to the plus-ends is mediated by kinesin-3, whereas that to the minus-ends is mediated by dynein, which enables long-range EE motility throughout the hyphal cell^8^.

Because motor proteins driven by ATPase activity support constant EE motility, cells constitutively consume an abundance of energy. Therefore, it has been speculated that EE motility is likely to have versatile physiological roles in living cells. Analyses in *U. maydis* have revealed that EE motility supports the “hitchhiking” of certain molecules, such as septin mRNAs and ribosomes^9,10^. Moreover, not only molecules but also organelles, such as peroxisomes (POs) and lipid droplets (LDs), can hitchhike via EE motility^11,12^. Furthermore, it has been suggested that EEs can transduce pathogenic cues from the infecting hyphal tip to the nucleus^13^. Thus, constantly moving EEs indeed have several biological roles. As a result, there might be as yet unidentified roles of EE motility in other filamentous fungi.

Recently, Hook, a linker protein between EEs and motor proteins, has been identified together with accessory proteins FHIP and FTS in both *U. maydis* and *A. nidulans* ^14–16^. When Hook is deleted, EE motility is abolished and the distribution of other organelles is also impaired: for example, POs and LDs accumulate at the hyphal tip, whereas endoplasmic reticulum (ER) is partially retracted to the basal region^11^. In *A. nidulans*, a linker protein, PxdA, that connects EEs and POs has also been identified^17^. The apical accumulation of POs and LDs in the absence of Hook can be explained by polar drift caused by the myosin motor^18^; however, the reason for ER retraction in the absence of EE motility is not clear. Furthermore, there are no detailed analyses of whether protein secretion is related to EE motility.

In this study, we have investigated the physiological roles of EE motility in *Aspergillus oryzae*, an industrially important fungus due to its property of abundant enzymatic protein secretion. By analyzing the disruptant of *Aohok1*, which encodes an ortholog of Hook, we confirmed that Δ*Aohok1* cells showed the same phenotypes of EE and PO distribution as observed in disruptants of *U. maydis* and *A. nidulans*. We further revealed that, without EE motility, formation of the apical secretory vesicle cluster Spitzenkörper was impaired, although the distributions of two other secretory organelles (ER and Golgi) were not affected. Moreover, we found that the transcript and protein levels of the *A. oryzae* major secretory protein α-amylase were significantly reduced in the absence of EE motility. Lastly, a lack of EE motility induced perturbation of conidial and sclerotial formations. Taken together, these results suggest that EE motility is crucial for abundant α-amylase production and proper cell differentiation in *A. oryzae*.

## Results

### Characterization of *A. oryzae* EEs

**Figure 1:**
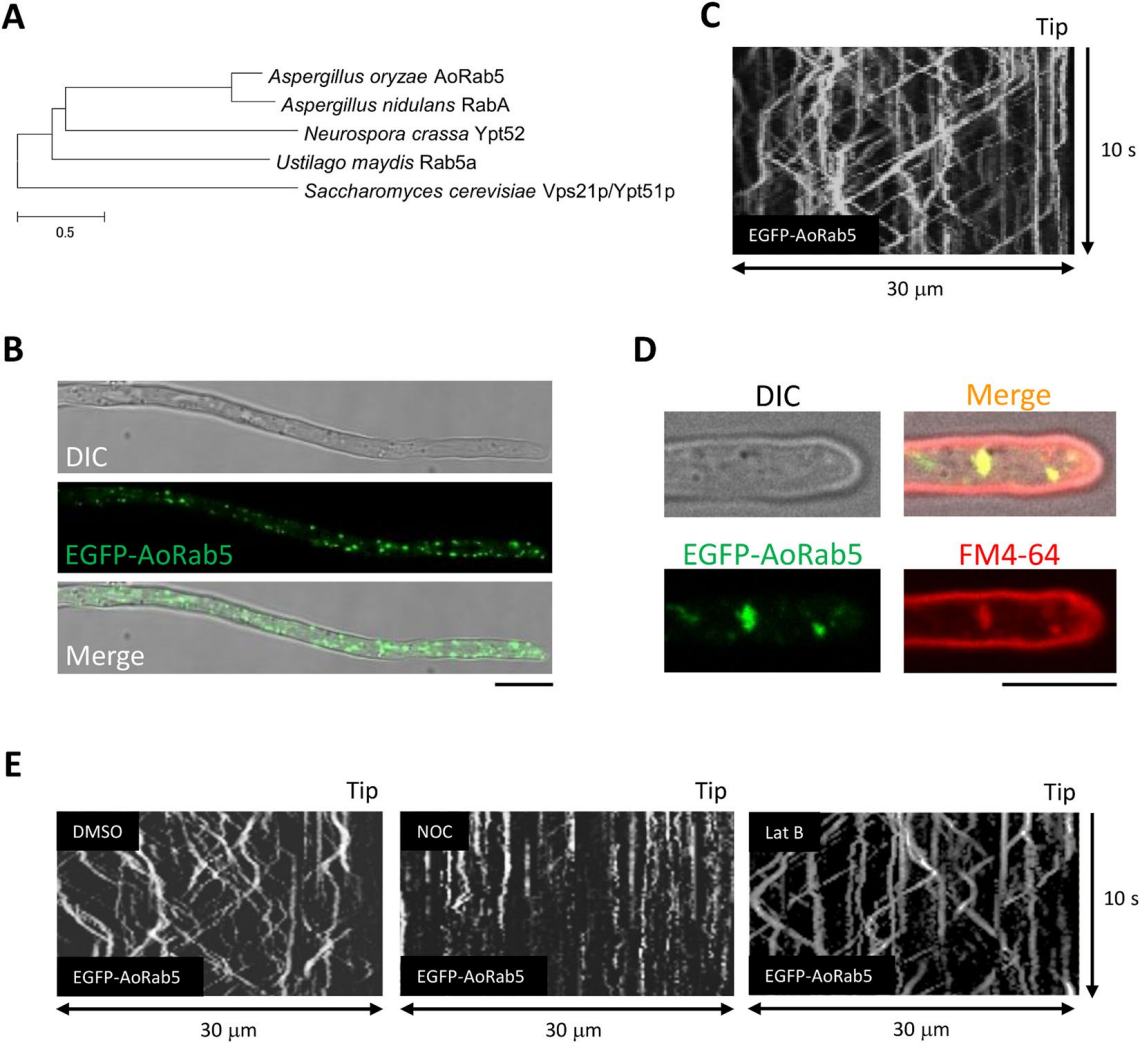
Early endosome motility visualized by the small GTPase Rab5 in *A. oryzae*. (**A**) Phylogenetic tree of Rab5 in fungi. (**B**) Subcellular distribution of EGFP-AoRab5 in an *A. oryzae* hypha. DIC, differential interference contrast. Scale bar, 10 µm. (**C**) Kymograph of EGFP-AoRab5 motility. (**D**) Colocalization of EGFP-AoRab5 and FM4-64-positive punctate structures. Images were taken approximately 10 min after FM4-64 staining. Scale bar, 5 µm. (**E**) Kymographs drawn from movies of EGFP-AoRab5 motility that were taken approximately 30 min after NOC, Lat B or control DMSO treatment.

To visualize the motility of EEs in *A. oryzae*, we first tried to establish an EE marker protein. In other model filamentous fungi, homologs of the small GTPase Rab5, which preferentially binds to EE membrane in its GTP form, have been characterized^5–7^. Thus, we conducted a BLAST search using these Rab5 homologs and identified a sole Rab5 homolog in *A. oryzae*, named AoRab5 (AO090003000619; Fig. 1A). An N-terminal EGFP fusion of AoRab5 showed dot-like structures distributed through the cell (Fig. 1B). In addition, EGFP-AoRab5 fluorescence exhibited characteristic, constant, long-range and bidirectional motility along a hypha (Fig. 1C; Supplementary Video 1). In a previous study, similar motility was observed for the plasma membrane purine transporter AoUapC tagged with EGFP, which was colocalized with the endocytic marker dye FM4-64, when endocytosis was induced in *A. oryzae* cells^19^. Therefore, to further identify the moving dots labelled with EGFP-AoRab5, we co-stained the cells with FM4-64. With a short chasing time of less than 10 min to observe motile EEs, we confirmed colocalization of EGFP-AoRab5 with FM4-64, indicating that the moving EGFP-AoRab5-labelled dots represent EEs (Fig. 1D). In general in filamentous fungi, MT and actin cytoskeletons are involved in membrane trafficking^18^. To examine whether EE motility is dependent on these cytoskeletons in *A. oryzae*, we treated cells with nocodazole (NOC) and latrunculin B (Lat B), polymerization inhibitors of MT and the actin cytoskeleton, respectively. As expected, treatment with NOC, but not Lat B, abolished EE motility, whereas the solvent DMSO control did not affect it (Fig. 1E; Supplementary Videos 2 and 3, DMSO and NOC). Taken together, the EGFP-AoRab5 construct enabled us to visualize MT-dependent long-range EE motility in *A. oryzae*.

### *A. oryzae* Hook and its deletion

**Figure 2:**
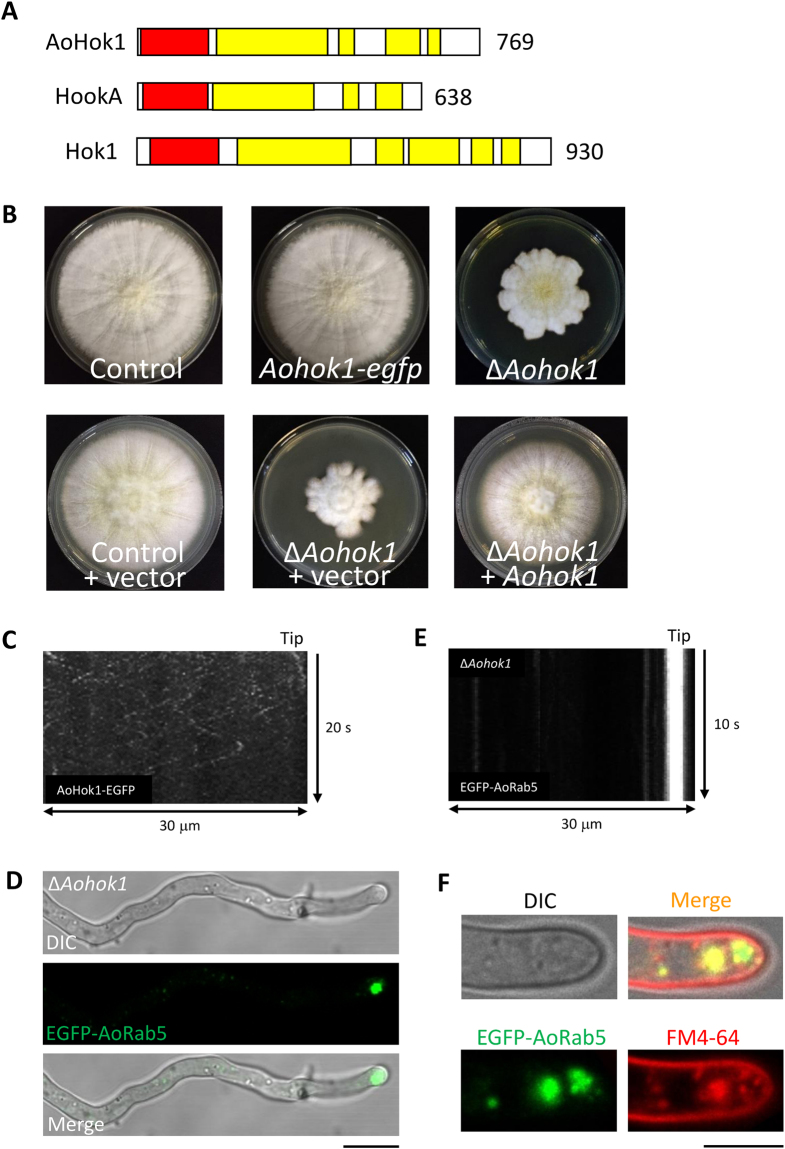
Characterization and deletion of *Aohok1*. (**A**) Schematic diagram of the predicted domain structure of *A. oryzae* AoHok1, *A. nidulans* HookA and *U. maydis* Hok1. Red and yellow boxes depict the Hook domain and coiled-coil domains, respectively. The number of amino acid residues is also indicated. (**B**) Strains of control, AoHok1-EGFP-expressing, Δ*Aohok1*, control introduced with vector, Δ*Aohok1* introduced with vector and Δ*Aohok1* complemented with *Aohok1* were grown on DPY plates at 30 °C for 7 days. (**C**) Kymograph of endogenously-expressed AoHok1-EGFP motility. (**D**) Subcellular localization of EGFP-AoRab5 in a Δ*Aohok1* hypha. DIC, differential interference contrast. Scale bar, 10 µm. (**E**) Kymograph of EGFP-AoRab5 motility in a Δ*Aohok1* hypha. (**F**) Δ*Aohok1* cells stained with FM4-64. Pictures were taken approximately 10 min after FM4-64 staining. Scale bar, 5 µm.

To generate an *A. oryzae* mutant impaired in EE motility, we attempted to identify a homolog of Hook. By searching the *A. oryzae* genome database for amino acid sequence matches to *A. nidulans* HookA and *U. maydis* Hok1, we identified AoHok1 (AO090012000999), which consists of 769 aa and harbors a domain structure similar to these orthologs (Fig. 2A; Fig. S1).

Next, to confirm whether AoHok1 exhibits motility like HookA and Hok1, we first generated a strain expressing *Aohok1-egfp* at the *Aohok1* locus and verified endogenous expression of the fusion protein. The *Aohok1-egfp* expressing strain showed similar growth to that of a control strain, suggesting the functionality of AoHok1-EGFP (Fig. 2B). Although the fluorescence signal was weak, AoHok1-EGFP exhibited bidirectional long-range motility (Fig. 2C; Supplementary Video 4). We then obtained a disruptant of *Aohok1*, confirmed by Southern blot analysis (Fig. S2). Similar to the *A. nidulans hookA* disruptant, we found that the *Aohok1* disruptant showed less growth; moreover, we observed that the colony had an abnormal shape (Fig. 2B), which was not reported in the *A. nidulans* study^16^. We introduced *Aohok1* gene into the *Aohok1* disruptant and found that in the resultant strain the growth and colony shape were restored, demonstrating that the phenotypes observed in the *Aohok1* disruptant were indeed caused by the deletion of *Aohok1* (Fig. 2B). In addition, we observed similar growth defects between control and Δ*Aohok1* cells at different temperatures and pH values, but the abnormal colony shape was only observed at 30 °C, pH 5.5 (Fig. S3A,B).

To confirm whether EE motility was abolished in Δ*Aohok1* cells, we introduced the above-established EE marker EGFP-AoRab5 into the disruptant. As expected, the motility of EEs was hardly observed and the organelles were clustered at the apical region in Δ*Aohok1* cells (Fig. 2D,E; Supplementary Video 5). We examined FM4-64 staining in the disruptant, which showed that endocytosis of FM4-64 was not defective and that internalized-FM4-64 was colocalized with EGFP-AoRab5 near the tip, confirming that the apical clustered structure still exhibited endocytic EE-like properties (Fig. 2F).

### *Aohok1* disruptant exhibits less sensitivity to osmotic and cell wall stresses

**Figure 3:**
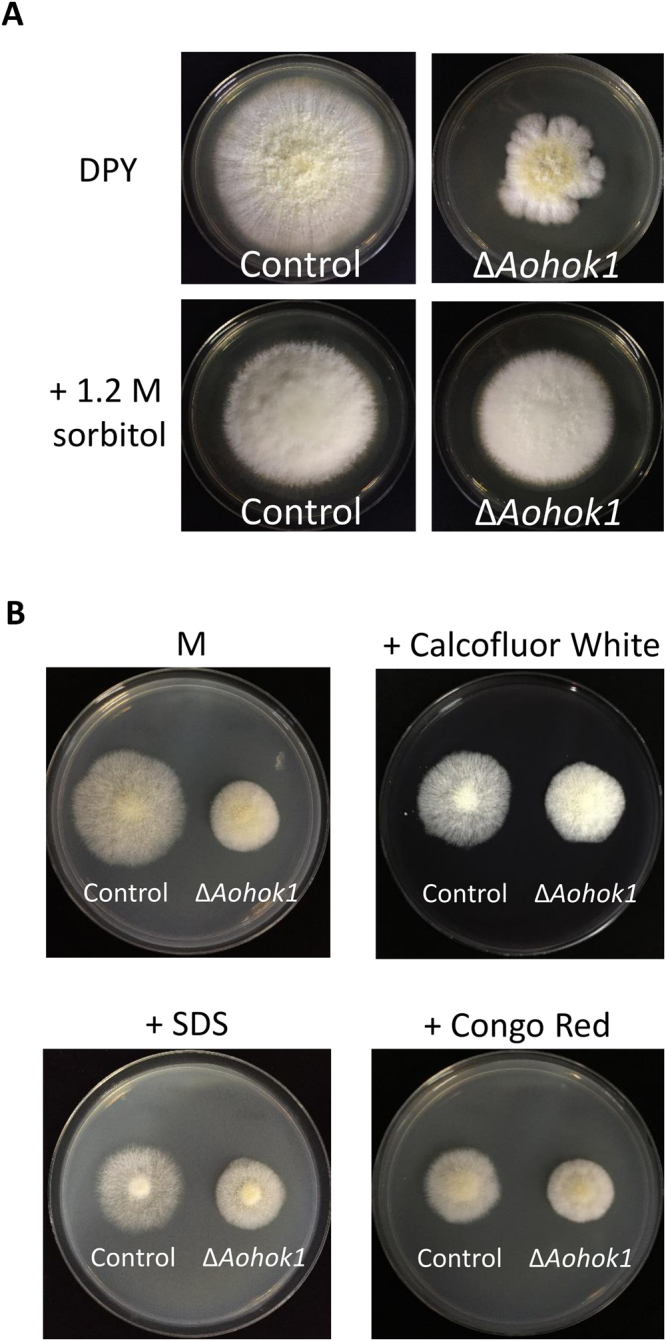
The *Aohok1* disruptant shows osmotic and cell wall stress tolerance. (**A**) Conidia of control and Δ*Aohok1* strains were inoculated onto DPY plates with or without 1.2 M sorbitol and incubated at 30 °C for 5 days. (**B**) Conidia of control and Δ*Aohok1* strains were inoculated onto M plates with or without Calcofluor White (300 µg/ml), SDS (90 µg/ml) or Congo Red (90 µg/ml) and incubated at 30 °C for 3 days.

When performing protoplast formation during the transformation procedure of *A. oryzae*, we noticed that Δ*Aohok1* cells were resistant to becoming protoplasts. Therefore, we wondered whether the cell wall structure of the *Aohok1* disruptant was abnormal. First, we visualized chitin, the major component of cell wall, by staining with Calcofluor White and found no obvious difference in the chitin content between cells of the control and Δ*Aohok1* strains (Fig. S4). Next, we tested osmotic stress and found that the *Aohok1* disruptant grew under a high sorbitol concentration to the same extent as the control strain (Fig. 3A). We also tested growth under a high salt condition, and found that the *Aohok1* disruptant showed less growth as compared with the control strain cultured with or without a high concentration of salt (Fig. S3C). Furthermore, to check cell wall stress sensitivity, we carried out growth tests using Calcofluor White, Congo Red and SDS. Even with these chemicals, the *Aohok1* disruptant exhibited normal growth, whereas the control strain showed sensitivity (Fig. 3B). Collectively, these results suggested that Δ*Aohok1* cells are less sensitive to osmotic and cell wall stresses.

### Subcellular distribution and function of POs in *Aohok1* disruptant

**Figure 4:**
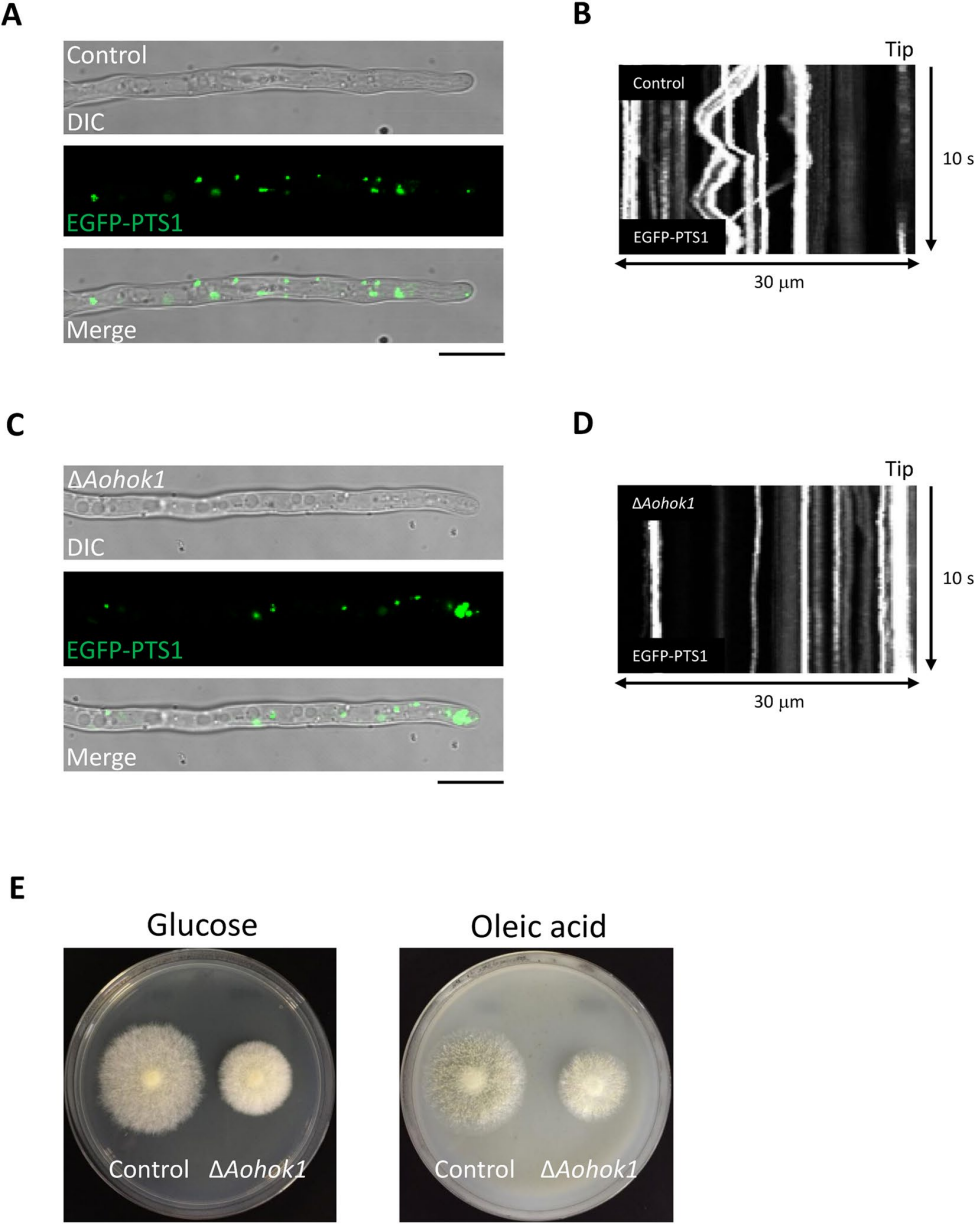
Subcellular distribution of peroxisomes and mycelial growth related to peroxisome function in the *Aohok1* disruptant. PO distribution in a hypha of the control (**A**) and Δ*Aohok1* (**C**) strains. Scale bars, 10 µm. Kymographs of PO motility in a hypha of the control (**B**) and Δ*Aohok1* (**D**) strains. (**E**) Control and Δ*Aohok1* strains were grown on plates containing either glucose or oleic acid as a sole carbon source at 30 °C for 3 days.

Because it has been reported that POs accumulate at the hyphal tip in both *U. maydis* Δ*hok1* and *A. nidulans* Δ*hookA* cells^11,16^, we investigated the distribution of POs in Δ*Aohok1* cells. POs were labeled with EGFP-PTS1, a previously established marker of POs in *A. oryzae* ^20^. In the control strain, POs were distributed throughout the cell and some populations exhibited motility (Fig. 4A,B; Supplementary Video 6). In Δ*Aohok1* hyphae, by contrast, POs were clustered at the apical region and lacked motility, suggesting that PO distribution is regulated by EE motility in *A. oryzae*, as well as in *U. maydis* and *A. nidulans* (Fig. 4C,D; Supplementary Video 7).

POs have a role in β–oxidation of fatty acids and a defect of this organelle function results in inability to grow on medium containing oleic acid as the sole carbon source^21^. To investigate whether the aberrant distribution of POs in Δ*Aohok1* affected their cellular function, we grew the Δ*Aohok1* strain on oleic acid plates. As compared with cells grown on glucose plates, those grown on oleic acid plates did not show any further defects, suggesting that an even distribution and motility are dispensable for PO function (Fig. 4E).

### Spitzenkörper organization is impaired in *Aohok1* disruptant

**Figure 5:**
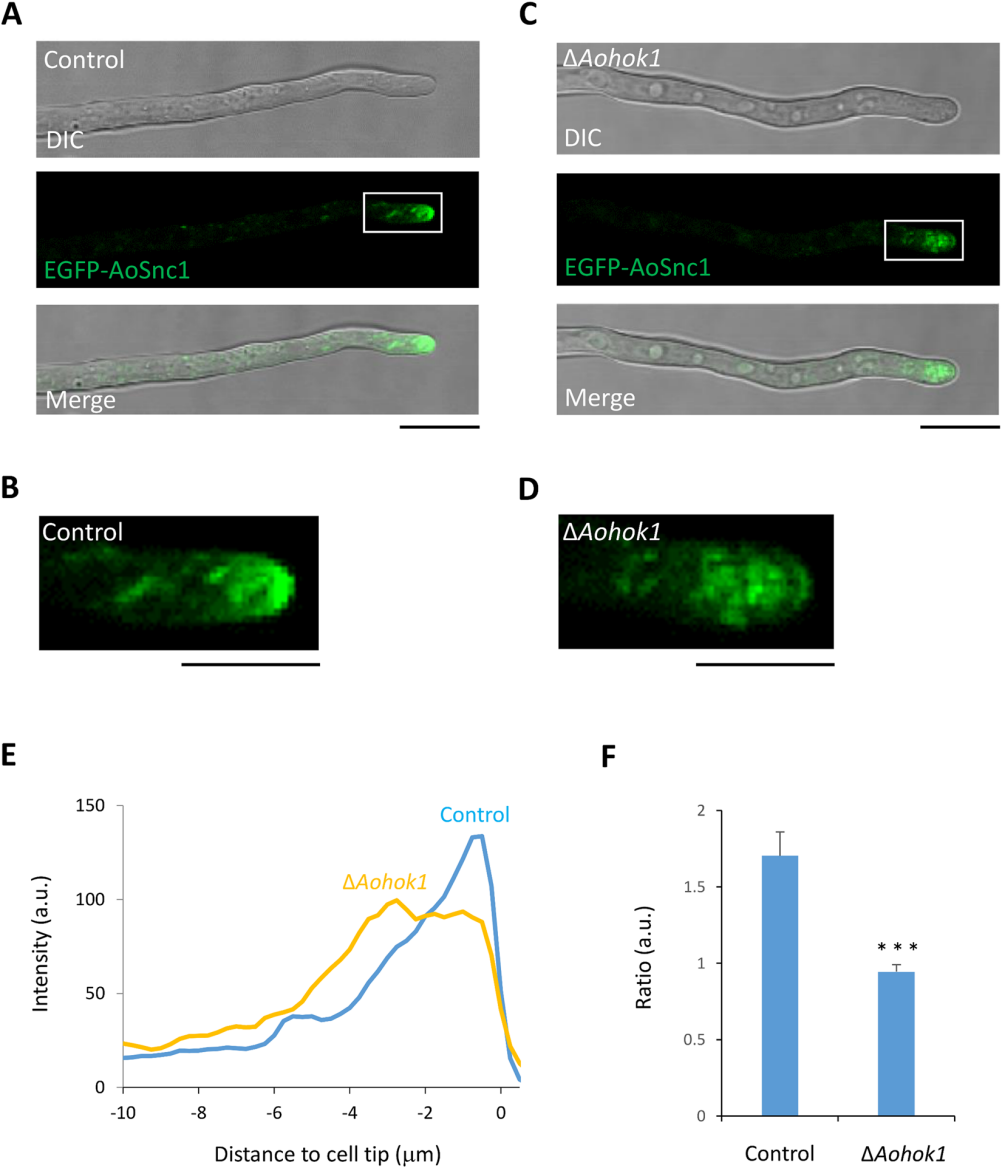
Apical clustering of secretory vesicles is impaired in the *Aohok1* disruptant. Secretory vesicle marker EGFP-AoSnc1 was visualized in control (**A**) and Δ*Aohok1* (**C**) strains. DIC, differential interference contrast. Scale bars, 10 µm. Enlarged images of the boxed areas in (**A**) and (**C**) are shown in (**B**) and (**D**), respectively. Scale bars, 5 µm. (**E**) Quantitative measurements of EGFP-AoSnc1 fluorescence intensity along hyphal cells of control and Δ*Aohok1* strains (n = 10). (**F**) Ratio of EGFP-AoSnc1 fluorescence intensity at the apical (0–2 µm) to the subapical (2–4 µm). ***Statistically significant difference at P < 0.001 (Student’s *t* test).

Next, we examined whether ER distribution was disordered in Δ*Aohok1* hyphae as it is in *U. maydis* Δ*hok1* cells^11^. Unexpectedly, ER distribution visualized by EGFP-AoSec22 was not largely affected in the Δ*Aohok1* strain as compared with the control strain (Fig. S4A,B). To analyze the protein secretory pathway, we visualized the Golgi apparatus by using its known marker EGFP-AoGos1^22^. We did not observe a conspicuous difference in Golgi distribution between the control and Δ*Aohok1* cells (Fig. S4C,D). We further examined secretory vesicles by using the marker EGFP-AoSnc1, which is mainly observed at the apical vesicle cluster Spitzenkörper^23^. In the control strain, EGFP-AoSnc1 was observed at the typical crescent-like structure of the tip, that is, the Spitzenkörper (Fig. 5A,B). In the *Aohok1* disruptant, by contrast, although EGFP-AoSnc1 was mainly localized near the tip region, the structure of the Spitzenkörper was more dispersed than in the control strain (Fig. 5C,D). These results suggested that secretory vesicles in the *Aohok1* disruptant were not properly targeted to the apical plasma membrane, where exocytosis predominantly occurs. Further quantitative analyses demonstrated that there was significantly less accumulation of secretory vesicles in the *Aohok1* disruptant than in the control strain (Fig. 5E,F).

### α-amylase production is reduced in *Aohok1* disruptant

**Figure 6:**
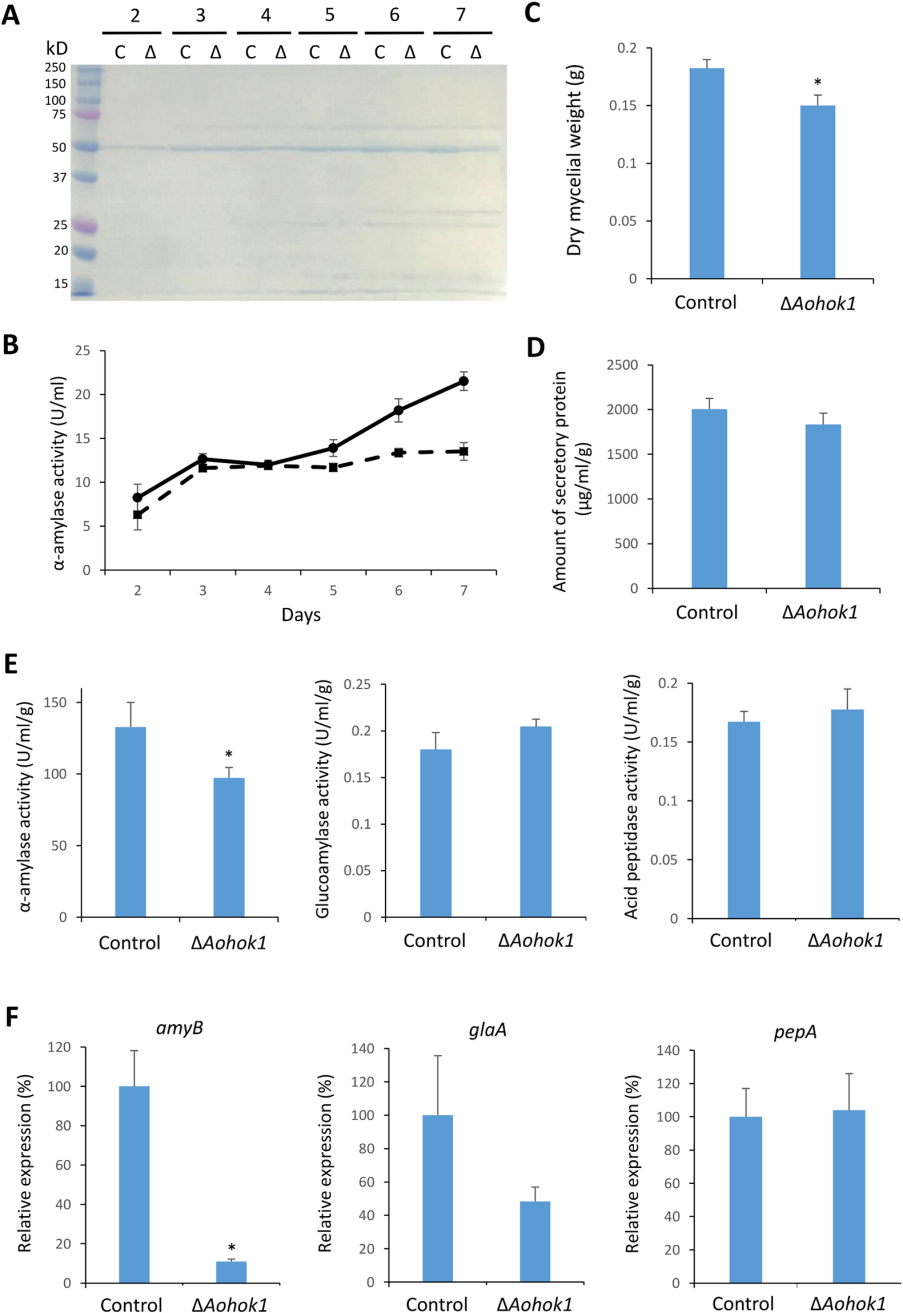
α-amylase production is defective in the *Aohok1* disruptant. (**A**) Culture supernatants of control (lanes C) and Δ*Aohok1* (lanes Δ) strains were taken at the indicated time points, analyzed by SDS-PAGE and stained with CBB. The band at ~50 kD band is known to be α-amylase. (**B**) α-amylase activity was measured in samples of culture supernatant taken on each day. Solid and dashed lines indicate control and Δ*Aohok1* strains, respectively. (**C**) Dry mycelial weight of each strain cultured after 7 days. (**D**) Total amount of secreted proteins in the culture supernatant of each strain after 7 days, normalized by dry mycelial weight. (**E**) Activities of α-amylase, glucoamylase and acid peptidase in culture supernatant from each strain cultured at 7 days, normalized by dry mycelial weight. (**F**) Relative expression levels of *amyB*, encoding α-amylase, *glaA*, encoding glucoamylase, and *pepA*, encoding acid peptidase, in cells of each strain cultured at 7 days, normalized by the expression level of *actA*, encoding actin. In (**C**,**E** and **F**) *statistically significant difference at P < 0.05 (Student’s *t* test). In (**B**–**F**), bars show mean ± SEM (n = 4).

Based on the microscopic analyses described above, we hypothesized that protein secretion might be defective in the absence of EE motility. *A. oryzae* abundantly secretes α-amylase into the culture medium, which can be easily detected by CBB staining on an acrylamide gel even without sample concentration. We measured the amount of secreted α-amylase by CBB staining and an activity assay. Both results consistently showed that less amount of α-amylase was secreted in the *Aohok1* disruptant than in the control strain, especially in the later phase of culture (Fig. 6A,B). The *Aohok1* disruptant grew less than the control strain in liquid culture as well as on plate culture (Fig. 6C). Although the difference was not significant, the total amount of secretory proteins was also slightly less in the *Aohok1* disruptant than in the control strain (Fig. 6D). Besides α-amylase, we investigated other secretory proteins glucoamylase and acid peptidase by measuring each enzymatic activity in the culture supernatant. We found that the activity of α-amylase, but not glucoamylase and acid peptidase, normalized by dry mycelial weight, was significantly less in the *Aohok1* disruptant (Fig. 6E).

Since in *U. maydis* EE motility is important for inducing transcription of effector genes^13^, we reasoned whether the transcript level of α-amylase might be perturbed without EE motility in *A. oryzae*. Indeed, consistent with activity data, we found that the transcript level of *amyB*, α-amylase-encoding gene, but not that of *glaA*, glucoamylase-encoding gene and *pepA*, acid peptidase-encoding gene, was significantly reduced in the *Aohok1* disruptant (Fig. 6F). Collectively, α-amylase production in the levels of both transcription and secretion was decreased in the *Aohok1* disruptant.

### Perturbation of cell differentiation in the absence of EE motility

**Figure 7:**
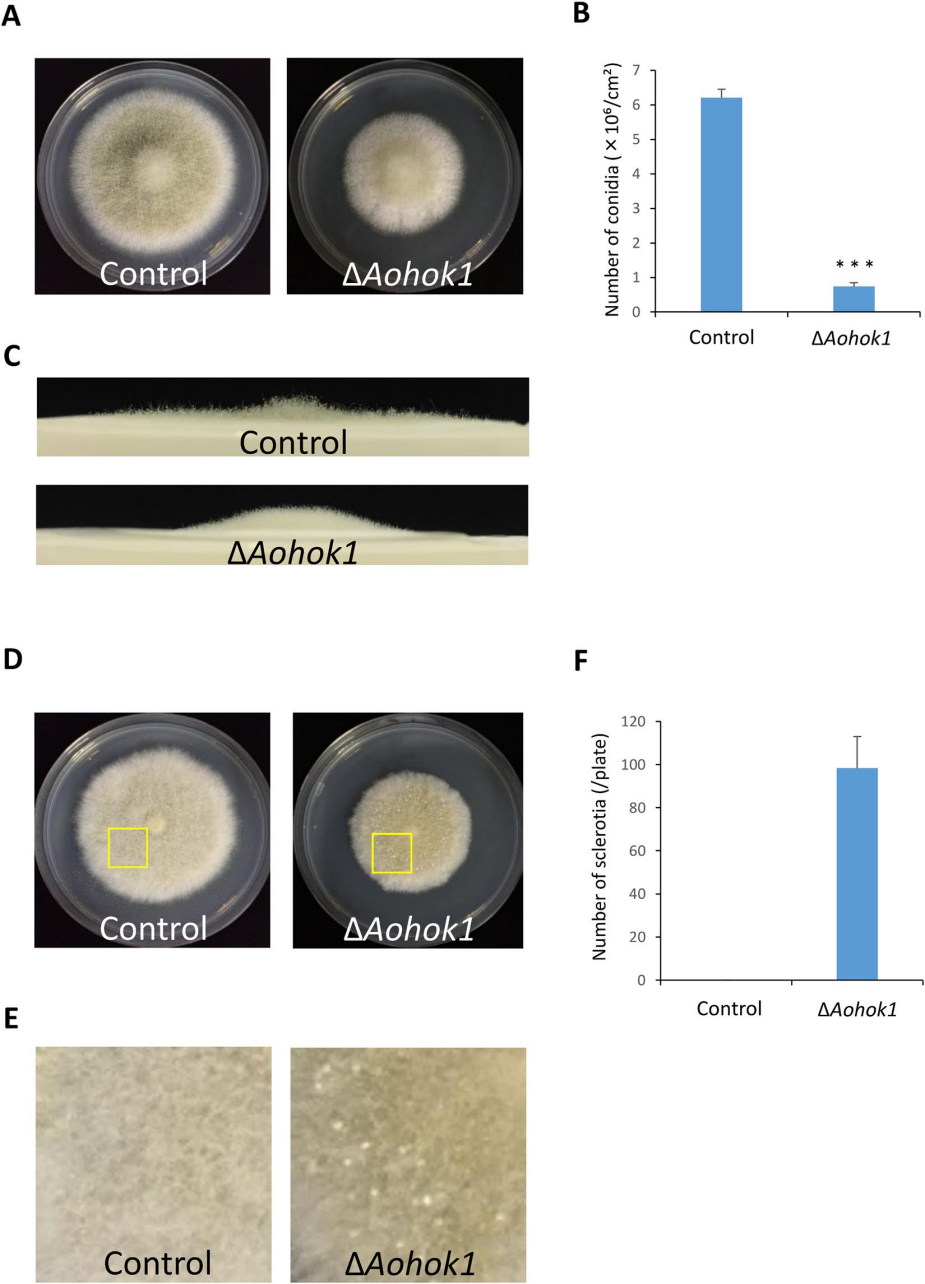
Formation of conidia and sclerotia is perturbed in the *Aohok1* disruptant. (A) Conidia of control and Δ*Aohok1* strains were inoculated onto PD plates and incubated at 30 °C for 5 days. (**B**) Quantitative measurements of conidial number were independently performed three times. Bars show mean ± SEM. ***Statistically significant difference at P < 0.001 (Student’s *t* test). (**C**) Side views of the mycelial plates shown in (**A**). Note that there were more aerial hyphae in Δ*Aohok1* than in the control. (**D**) Conidia of control and Δ*Aohok1* strains were inoculated onto M plates and incubated at 30 °C for 8 days. (**E**) Enlarged images taken from the boxed areas in (**D**). Note that white dot-like structures, sclerotia, were seen in the mycelium of Δ*Aohok1*, but not the control. (**F**) Quantitative measurements of sclerotial number were independently performed three times. Bar shows mean ± SEM.

Motility of EEs is thought to be crucial for signal transduction in filamentous fungi^24^. In the corn smut fungus *U. maydis*, a lack of EE motility results in attenuated virulence caused by less expression of effector genes, the products of which are essential for pathogenicity^13^. In *A. oryzae*, cell differentiation, including conidial and sclerotial formation, is regulated by specific components^25–27^. Therefore, we reasoned whether an absence of EE motility might affect the formation of conidia or sclerotia. First, to investigate whether EE motility was responsible for conidiation, we tested growth on PD plates where *A. oryzae* normally makes abundant conidia. The *Aohok1* disruptant produced fewer conidia as compared with the control strain (Fig. 7A,B). Microscopic observation revealed that there was no obvious difference in conidial morphology between the control and Δ*Aohok1* strains (Fig. S6). Generally, mutants defective in conidial formation, such as autophagy mutants, cannot make aerial hyphae^28^. However, the *Aohok1* disruptant produced even more aerial hyphae than the control strain (Fig. 7C). Next, we examined the formation of sclerotia, mycelial structures in a sexual-like stage, that is normally suppressed in the wild-type background strain of *A. oryzae*. We found that the *Aohok1* disruptant generated sclerotia, whereas the control strain did not (Fig. 7D–F). Taken together, these findings showed that abolishing EE motility induced abnormal cell differentiation in the *Aohok1* disruptant.

## Discussion

Here, we have demonstrated physiological roles of long-range MT-dependent EE motility in *A. oryzae*. Owing to the nature of the filamentous fungal elongated cell shape, this long-range motility is thought to be crucial for delivering intracellular molecules to their proper localization. For example, recent studies have revealed that cell wall synthases and acyl-CoA binding protein are MT-dependent cargo proteins^29,30^. In intracellular membrane trafficking, EEs are convenient porters for delivering molecules due to their constant bidirectional motility. Not only specific molecules, but also certain organelles, such as PO and LD, are reliant on moving EEs for their position inside the cell^11^. In particular, ER and endosome contact has been reported to be involved in several cellular functions in other eukaryotes^31–33^. Furthermore, the interplay of other organelles, such as LD and ER, has physiological roles^34^. In *A. oryzae*, as in *U. maydis* and *A. nidulans*, a lack of EE motility resulted in an apical PO cluster without motility. However, this abnormal PO distribution did not lead to a dysfunctional phenotype. The physiological importance of the motility and subcellular distribution of PO needs further investigation.

Unexpectedly, subcellular distribution of the ER and Golgi was not affected even in the absence of EE motility in *A. oryzae*. Localization of the rough ER might be dependent on that of nuclei, which is supported by the organization of cytoplasmic MTs. Similarly, because nuclear localization was not impaired in the *hookA* disruptant, ER localization might not be affected in *A. nidulans* ^16^. In *U. maydis*, however, the ER is partially retracted from the tip region in the absence of EE motility^11^. This difference in ER distribution between *A. oryzae* and *U. maydis* might arise because the former is a multinuclear fungus, whereas the latter is mononuclear, at least under the study conditions. In addition, a lack of EE motility did not largely affect the localization of Golgi bodies in *A. oryzae*. A recent report on *Saccharomyces cerevisiae* suggests that there may be a mechanism to regulate Golgi localization inside cells^35^. Because the ER and Golgi are crucial organelles for protein secretion, *A. oryzae* might have established mechanisms to distribute these organelles throughout the cell, independent of EE motility.

**Figure 8:**
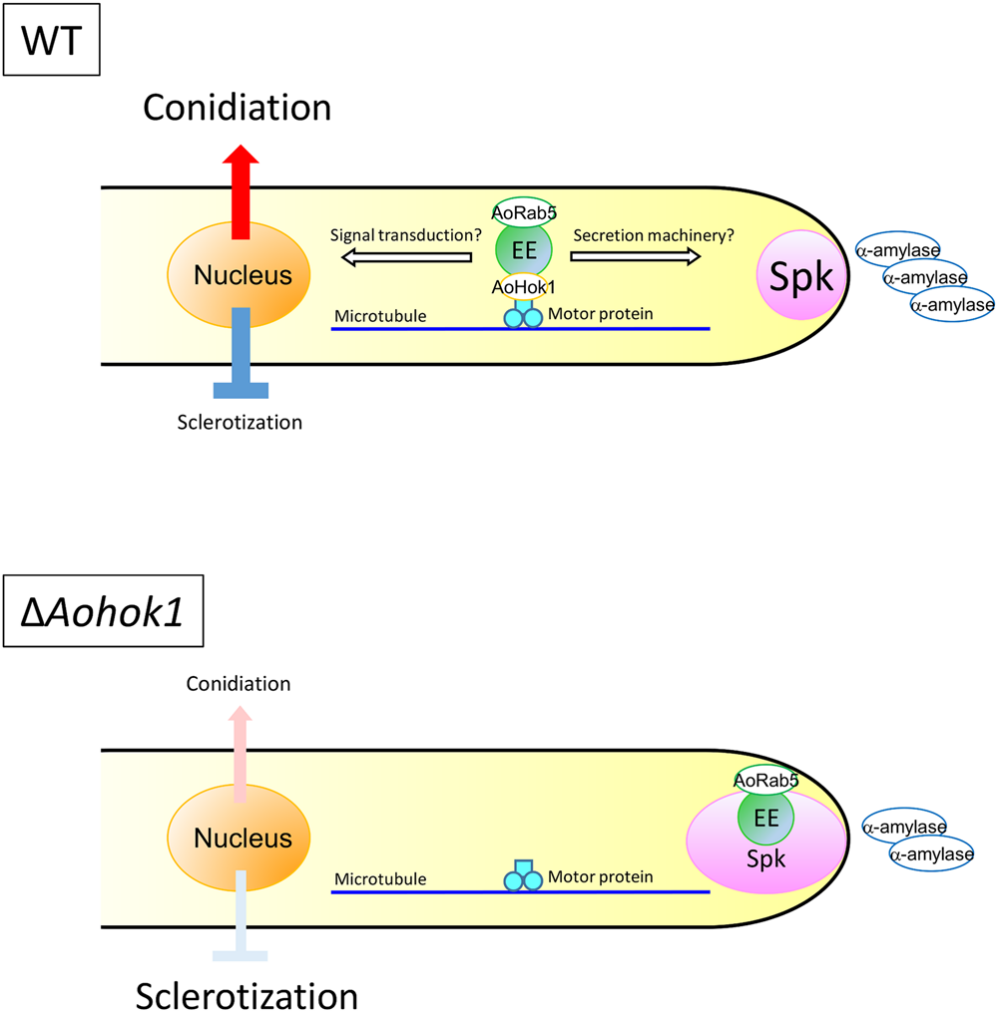
Model of early endosome motility in apical protein secretion, conidiation and sclerotization. Constant EE motility supports efficient apical protein secretion and maintains conidiation upregulation under dark conditions and sclerotization downregulation under nutrient-limited conditions. In Δ*Aohok1* cells, EEs are clustered at the hyphal tip region. In the absence of EE motility, transport of secretion machinery to the tip and signaling molecules to the nucleus might be deficient, resulting in less protein secretion and conidiation, but in derepression of sclerotization. Spk, Spitzenkörper.

We found that EE motility is required for efficient secretion of the *A. oryzae* major protein α-amylase, which is thought to be transported through ER and Golgi and mainly secreted from the hyphal tip^36–38^. A lack of EE motility resulted in less α-amylase secretion, probably due to disorganization of the Spitzenkörper, rather than to perturbed distribution of the ER and Golgi. A simple explanation for this phenotype is that EEs transport some components required for apical protein secretion, including the v-SNARE AoSnc1 (Fig. 8). The mechanism that regulates Spitzenkörper organization is not fully understood^39^; thus, how EE motility is involved in this process needs further examination. Furthermore, we revealed that the transcript level of α-amylase-encoding gene *amyB* was reduced in the absence of EE motility. Thus, there might be certain regulation mechanisms of α-amylase production in the levels of both transcription and secretion, where EE motility is involved in. We also observed the abnormal colony morphology of *Aohok1* disruptant on optimized rich medium. Considering that protein secretion occurs abundantly in such nutrient-rich condition, EE motility might have another supportive role in polarity maintenance and growth.

In *U. maydis*, it has been reported that EE motility is crucial for fungal infection into plant cells^13^. EEs probably transport signaling molecules to convey them to the nucleus, but specific molecules have not yet been identified, although a MAPK has been found to be a negative regulator. In *A. oryzae*, removal of EE motility perturbed cell differentiation, producing fewer conidia and more sclerotia, and forming higher aerial hyphae. These are phenocopies of the overexpression of AoAtg1, which functions in both autophagy and the cytoplasm-to-vacuole targeting (Cvt) pathway^40^. Therefore, it is possible that autophagy and/or Cvt activity might be increased in the *Aohok1* disruptant, although this needs further investigation. Cell differentiation should be tightly regulated, and we speculate the constant EE motility might be involved in conveying environmental signals to the nucleus (Fig. 8). Some of the molecular components involved in conidiation and sclerotization are known in *A. oryzae* ^25–27^. Whether the transcripts of such components related to cell differentiation are perturbed in the absence of EE motility will be investigated in future studies.

Besides its use in traditional industrial fermentation, *A. oryzae* is an excellent host for producing valuable materials, such as pharmaceutical proteins and secondary metabolites^41,42^. It is worth mentioning that some secondary metabolites are secreted through intracellular membrane trafficking^43^. Indeed, in *A. nidulans*, endosomes are involved in melanin production^44^. Each secondary metabolite seems to undergo a different secretion pathway through discrete organelles. Given that EE motility is not essential for cell growth, it might be important for regulating the production of secondary metabolites. Moreover, considering that *A. oryzae* is grown by solid-state culture in traditional fermentation, it is tempting to analyze physiological roles of EE motility in such culture condition. Because grains, such as rice and barley, are used in solid-state culture, the molecular mechanisms regulating fungal-plant interaction can also be dissected. For more efficient production of valuable materials using *A. oryzae* cells, further detailed investigation on the underlying molecular mechanisms and physiological roles of EE motility will be needed.

## Methods

### DNA cloning and strain construction

The *A. oryzae* strains and primers used in this study are listed in Supplementary Tables 1 and 2, respectively. Genomic DNA of the wild-type *A. oryzae* strain RIB40 was used as the template for common DNA cloning^45^. Control strains for each experiment were used to have the same auxotrophy with Δ*Aohok1* background strains. For visualizing EGFP-tagged proteins, an expression vector pgPaegSmnD, incorporating P*amyB*, *egfp*, SmaI site and *niaD* marker, was constructed. To prepare its vector sequence, inverse PCR was performed by using PrimeSTAR MAX DNA polymerase (Takara), primers SH1 and SH2, and pgAUEN as a template. The DNA sequence of *egfp* with SmaI site was amplified as an insert by using PrimeSTAR GXL DNA polymerase (Takara), primers YT13 and YT30 and pgAUEN as a template. These DNA fragments of vector and insert were ligated by In-Fusion reaction (Takara), resulting in pgPaegSmnD. For preparing pgPaegR5nD, pgPaegPOnD, pgPaegS22nD, pgPaegGOSnD and pgPaegSnnD, DNA sequences of *Aorab5*, *egfp-SKL*, *Aosec22*, *Aogos1* and *Aosnc1* were amplified by PCR using PrimeSTAR GXL DNA polymerase, RIB40 genomic DNA and primer sets YT26 and YT31, YT13 and YT28, YHK160 and YHK161, YHK146 and YHK147, and YHK119 and YHK120, respectively.

To generate endogenously expressing *Aohok1-egfp* construct, first we created pgegTasC, harboring *egfp*, T*amyB* and *AosC*. Approximately 1 kb each of *Aohok1* ORF without stop codon and downstream were amplified by PCR using PrimeSTAR GXL DNA polymerase, RIB40 genomic DNA and primer sets YT104 and YT105 and YT106 and YT107, respectively. The amplified products and the *egfp-*T*amyB-AosC* sequence, prepared from pgegTasC by NotI digestion, were ligated by In-Fusion reaction, resulting in pgHkegTasC. This plasmid was digested by NotI, yielding *Aohok1-egfp-*T*amyB-AosC*, which was introduced into the *Aohok1* locus by homologous recombination of *A. oryzae* transformation.

To generate a construct for *Aohok1* deletion, approximately 1 kb of both the upstream and downstream regions of *Aohok1* ORF were amplified by PCR using RIB40 genomic DNA as the template and primer sets YT5 and YT6, and YT7 and YT8, respectively. We generated a linear DNA cassette containing the *AosC* marker in-between the *Aohok1* upstream and downstream regions was conducted. The DNA cassette was transformed into the *A. oryzae* strain NSlD1 as described previously^46^. The transformants obtained were confirmed by Southern blot analysis using a probe that was prepared with primers YT44 and YT45. Each of the EGFP constructs described above was introduced into the control NSlDS1 and Δ*Aohok1* strains. For complementation of *Aohok1*, approximately 1.5 kb of the upstream region of *Aohok1* ORF, 2.7 kb of *Aohok1* ORF and 0.5 kb of the downstream region of *Aohok1* ORF were amplified by PCR using RIB40 genomic DNA as the template and primer sets YT149 and YT150. The amplified DNA was incorporated into the expression vector containing *niaD* marker, which was prepared using pgPaegR5nD as a template and primers YT151 and YT152.

### Culture media

Czapek-Dox (CD) (0.3% NaNO_3_, 0.2% KCl, 0.1% KH_2_PO_4_, 0.05% MgSO_4_·7H_2_O, 0.002% FeSO_4_·7H_2_O and 2% glucose, pH 5.5) and Minimal (M) (0.2% NH_4_Cl, 0.1% (NH_4_)_2_SO_4_, 0.05% KCl, 0.05% NaCl, 0.1% KH_2_PO_4_, 0.05% MgSO_4_·7H_2_O, 0.002% FeSO_4_·7H_2_O and 2% glucose, pH 5.5) media were used for standard growth. For growth tests, dextrin-polypeptone-yeast extract (DPY; 2% dextrin, 1% polypeptone, 0.5% yeast extract, 0.5% KH_2_PO_4_ and 0.05% MgSO_4_·7H_2_O) and potato dextrose (PD; Nissui) plates were used.

### Fluorescence microscopy

For microscopic observation, we exploited a TCS SP8 inverted microscope (Leica) equipped with a 100× objective lens (1.40 numerical aperture), a HyD detector, an FOV scanner, and 488 nm and 561 nm argon lasers for EGFP and FM4-64 fluorescence, respectively. Image data were acquired by using LAS X software (Leica). Kymograph and fluorescence intensity analyses were performed via the respective functions of MetaMorph software (Molecular Devises). For observation culture, approximately 10^5^ conidia of each strain were inoculated with 100 µl of an appropriate medium in a glass-base dish (Iwaki) and incubated at 30 °C for around 20 h. Staining with FM4-64 and Calcofluor White was performed as described previously^23^. Inhibitor treatments using stocks of nocodazole (NOC; Sigma) and latrunculin B (Lat B; Calbiochem) were carried out as described previously^19,47^. NOC and Lat B were used at a final concentration of 100 µg/ml and 100 µM from stock solutions at a concentration of 10 mg/ml and 10 mM, respectively, suspended in DMSO.

### Growth tests

A conidial suspension of the control or Δ*Aohok1* strain (~10^3^ or 10^5^/10 µl) was spotted onto each medium plate and incubated at 20 °C, 30 °C or 37 °C for 3 to 8 days. To test cell wall stress tolerance, either 300 µg/ml of Calcofluor White (Sigma), 90 µg/ml of SDS (Nacalai tesque) or 90 µg/ml of Congo Red (Nacalai tesque) was added to M agar plates.

### Protein secretion analysis

Approximately 10^5^ conidia of the control or Δ*Aohok1* strain was inoculated into 20 ml of DPY medium in a 100 ml Erlenmeyer flask and cultured at 30 °C for up to 7 days. After 7 days culture, the dry mycelial weight harvested from each culture was recorded. At each day point, 100 µl of each culture supernatant was collected for analyses of SDS-PAGE, α-amylase activity and total amount of secreted protein. A gel of 12% acrylamide was used for SDS-PAGE analysis and stained with CBB EzStain AQua (Atto) to visualize major secretory protein α-amylase at around 50 kD. Activities of α-amylase, glucoamylase and acid peptidase were analyzed by using each enzyme measuring kit (Kikkoman). Total protein was determined by Bradford dye reagent (Takara) according to the manufacturers’ instructions.

### Quantitative RT-PCR analysis

Total RNA was extracted from cells of each strain cultured in 20 ml of DPY medium for 7 days. cDNA was synthesized using SuperPrep Cell Lysis & RT Kit for qPCR (Toyobo) according to the manufacturer’s instructions. Quantitative RT-PCR (qRT-PCR) analysis was performed using Thunderbird SYBR qPCR Mix (Toyobo) and a Thermal Cycler Dice Real Time System TP-800 instrument (Takara) essentially as described previously^48^. Each cDNA sample was analyzed in triplicate. The transcript level was analyzed using primers as follows (sequences are summarized in Supplementary Table 2): YHK188 and YHK189 for *amyB* (AO090120000196); YHK190 and YHK191 for *glaA* (AO090010000746); and YHK192 and YHK193 for *pepA* (AO090120000474). The expression level of each gene was normalized to that of *actA* (AO090701000065) using primers YHK194 and YHK195.

### Bioinformatic analysis

To identify sequences of AoRab5 and AoHok1, we performed BLAST searches of the database of AspGD (http://www.aspgd.org/). A phylogenetic tree for AoRab5 and its orthologs was generated by using the program MEGA6. The amino acid sequences of AoHok1, HookA and Hok1 were aligned with CLUSTAL W (http://www.genome.jp/tools/clustalw/). Prediction of functional domains and coiled-coil regions in AoHok1, HookA and Hok1 was carried out by using the programs Pfam (http://pfam.xfam.org/) and COILS (http://www.ch.embnet.org/software/COILS_form.html), respectively.

## Electronic supplementary material

Supplementary video S1

Supplementary video S2

Supplementary video S3

Supplementary video S4

Supplementary video S5

Supplementary video S6

Supplementary video S7

Supplementary information
