# Supplementary material for: Early endosome motility mediates α-amylase production and cell differentiation in Aspergillus oryzae
Source: Sci Rep. 2017 Nov 17;7:15757. doi: 10.1038/s41598-017-16163-1 (PMC5693997; doi:10.1038/s41598-017-16163-1)
Supplement: Supplementary file 8 — Supplementary information [file 41598_2017_16163_MOESM8_ESM.pdf]

Supplementary Information

Early endosome motility mediates α-amylase production and cell differentiation in *Aspergillus oryzae*

Yusuke Togo, Yujiro Higuchi*, Yoshinori Katakura, Kaoru Takegawa

Department of Bioscience and Biotechnology, Faculty of Agriculture, Kyushu University, 6-10-1 Hakozaki, Fukuoka 812-8581, Japan

*Corresponding author. Tel/Fax: +81 92 642 2851, E-mail address: y.higuchi@agr.kyushu-u.ac.jp

Word count: Abstract, 200; Main, 3161; Methods, 1104.

Number of figures: Main, 8; Supplementary, 6.

Number of tables: Supplementary, 2.

Number of videos: Supplementary, 7.

Supplementary figure legends

Supplementary figure 1. Sequence alignment of AoHok1 and its orthologs.

Shown is alignment of the amino acid sequences of *A. oryzae* AoHok1, *A. nidulans* HookA and *U. maydis* Hok1.

Supplementary figure 2. Southern blot analysis of the *Aohok1* disruptant.

A scheme for Southern blot analysis using EcoRV is shown.

Supplementary figure 3. Growth tests in different culture conditions.

Conidia of control and Δ*Aohok1* strains were inoculated onto DPY plates at different temperatures (A), pH (B) and with or without 1 M NaCl.

Supplementary figure 4. Calcofluor White staining in the *Aohok1* disruptant.

To visualize chitin in cell wall, Calcofluor White staining was performed in control and Δ*Aohok1* strains. Scale bar, 10 µm. DIC, differential interference contrast.

Supplementary figure 5. Subcellular distribution of the ER and Golgi apparatus in the *Aohok1* disruptant.

An ER marker (EGFP-AoSec22) was observed in control (A) and Δ*Aohok1* (B) strains. The Golgi apparatus was visualized with EGFP-AoGos1 in control (C) and Δ*Aohok1* (D) strains. Scale bars, 10 µm (A and B), 5 µm (C and D). DIC, differential interference contrast.

Supplementary figure 6. Morphology of conidia in the *Aohok1* disruptant.

Conidial morphology was observed in control and Δ*Aohok1* strains. Scale bar, 5 µm.

DIC, differential interference contrast.

Supplementary Table 1. Strains used in this study.

Strain	Genotype	Reference
RIB40	Wild-type	
NSlD1	*niaD*$^-$ *sC*$^-$ *adeA*$^-$ Δ*argB* Δ*ligD*::*argB* Δ*pyrG*::*adeA* *pyrG*	46
NSlDS1	*niaD*$^-$ *sC*$^-$ *AosC* *adeA*$^-$ Δ*argB* Δ*ligD*::*argB* Δ*pyrG*::*adeA* *pyrG*	This study
NSlDSN1	*niaD*$^-$ *niaD* *sC*$^-$ *AosC* *adeA*$^-$ Δ*argB* Δ*ligD*::*argB* Δ*pyrG*::*adeA* *pyrG*	This study
GRab5	*niaD*$^-$ (P*amyB*-*egfp*-*Aorab5* *niaD*) *sC*$^-$ *AosC* *adeA*$^-$ Δ*argB* Δ*ligD*::*argB* Δ*pyrG*::*adeA* *pyrG*	This study
AoHok1G	*niaD*$^-$ *sC*$^-$ *Aohok1*-*egfp*::*AosC* *adeA*$^-$ Δ*argB* Δ*ligD*::*argB* Δ*pyrG*::*adeA* *pyrG*	This study
Δ*Aohok1*	*niaD*$^-$ *sC*$^-$ Δ*Aohok1*::*AosC* *adeA*$^-$ Δ*argB* Δ*ligD*::*argB* Δ*pyrG*::*adeA* *pyrG*	This study
ΔA*ohok1*N	*niaD*$^-$ *niaD* *sC*$^-$ Δ*Aohok1*::*AosC* *adeA*$^-$ Δ*argB* Δ*ligD*::*argB* Δ*pyrG*::*adeA* *pyrG*	This study
Δ*Aohok1_Aohok1*	*niaD*$^-$ (P*Aohok1*-*Aohok1*-T*Aohok1* *niaD*) *sC*$^-$ Δ*Aohok1*::*AosC* *adeA*$^-$ Δ*argB* Δ*ligD*::*argB* Δ*pyrG*::*adeA* *pyrG*	This study
Δ*Aohok1*_GRab5	*niaD*$^-$ (P*amyB*-*egfp*-*Aorab5* *niaD*) *sC*$^-$ Δ*Aohok1*::*AosC* *adeA*$^-$ Δ*argB* Δ*ligD*::*argB* Δ*pyrG*::*adeA* *pyrG*	This study

GPO1	*niaD*$^-$ (P*amyB-egfp-SKL niaD*) *sC*$^-$ *AosC adeA*$^-$ Δ*argB* Δ*ligD*::*argB* Δ*pyrG*::*adeA pyrG*	This study
Δ*Aohok1*_GPO1	*niaD*$^-$ (P*amyB-egfp-SKL niaD*) *sC*$^-$ Δ*Aohok1*::*AosC adeA*$^-$ Δ*argB* Δ*ligD*::*argB* Δ*pyrG*::*adeA pyrG*	This study
GSec22	*niaD*$^-$ (P*amyB-egfp-Aosec22 niaD*) *sC*$^-$ *AosC adeA*$^-$ Δ*argB* Δ*ligD*::*argB* Δ*pyrG*::*adeA pyrG*	This study
Δ*Aohok1*_GSec22	*niaD*$^-$ (P*amyB-egfp-Aosec22 niaD*) *sC*$^-$ Δ*Aohok1*::*AosC adeA*$^-$ Δ*argB* Δ*ligD*::*argB* Δ*pyrG*::*adeA pyrG*	This study
GGos1	*niaD*$^-$ (P*pgkA-egfp-Aogos1 niaD*) *sC*$^-$ *AosC adeA*$^-$ Δ*argB* Δ*ligD*::*argB* Δ*pyrG*::*adeA pyrG*	This study
Δ*Aohok1*_GGos1	*niaD*$^-$ (P*pgkA-egfp-Aogos1 niaD*) *sC*$^-$ Δ*Aohok1*::*AosC adeA*$^-$ Δ*argB* Δ*ligD*::*argB* Δ*pyrG*::*adeA pyrG*	This study
GSnc1	*niaD*$^-$ (P*pgkA-egfp-Aosnc1 niaD*) *sC*$^-$ *AosC adeA*$^-$ Δ*argB* Δ*ligD*::*argB* Δ*pyrG*::*adeA pyrG*	This study
Δ*Aohok1*_GSnc1	*niaD*$^-$ (P*pgkA-egfp-Aosnc1 niaD*) *sC*$^-$ Δ*Aohok1*::*AosC adeA*$^-$ Δ*argB* Δ*ligD*::*argB* Δ*pyrG*::*adeA pyrG*	This study

Supplementary Table 2. Primers used in this study.

Name	Sequence (5’ to 3’)
SH1	CATAGTTGATAATTCACTGGCCGTCG
SH2	CTGTGGGGTTTATTGTTCAGAGAAGG
YHK119	GAGCTGTACAAGCCCATGTCTGAACAACCTTACGAC
YHK120	TTATCAACTATGCCCTTAATGCTTAGTAGCGACAAC
YHK146	GAGCTGTACAAGCCCATGGCTGCCTCAACAGGTAC
YHK147	TTATCAACTATGCCCTTATCGGAAAAAAAGCAGCATCAAAAAGCAG
YHK160	GAGCTGTACAAGCCCATGGTCAAATCGACTCAAATAGC
YHK161	TTATCAACTATGCCCTTAGAAGAAGCGCAACCAAATG
YHK188	CCTGGGCACATTCGTCGAGA
YHK189	AATGCTGCGACGTTCTTGGC
YHK190	CGGTTCCTCGTGCCCCTATT
YHK191	GATGTCTTTGCCCGATCGCC
YHK192	TCCCCGCAATCTACAGCAGG
YHK193	GCCGCCAATGTTGACAGGAG
YHK194	TCCAACCGTGAGAAGATGACCC
YHK195	GAATCCAGAACGATACCGGTGGTA
YT5	TATCAACGCGGCCGCCAAAGGATTCTGTACTGACTTGACAC
YT6	ACTAAATCGCGGCCGTTGGCCTAAGAGGCAGTTG
YT7	GTTCCTTGGGCGGCCGATGACTGGTACGTCCTAATTCGAAGAC
YT8	CACCATAGCGGCCGCCCCCGAGGATGTCAAGCATTTTC
YT13	CAATAAACCCCACAGATGGTGAGCAAGGGCGAGGAGC

YT26	GAGCTGTACAAGCCCATGTCTGAGTCAACTAGCACAAATAC
YT28	GAATTATCAACTATGTTAAAGCTTGCTCTTGTACAGCTCGTCCATGCCGTGAG
YT30	GAATTATCAACTATGCCCGGGCTTGTACAGCTCGTCCATGCCGTGAG
YT31	GAATTATCAACTCCCTTAACAAGCACAACCTTCTTTGGC
YT44	GATGACTGGTACGTCCTAATTCGAAGACTGGC
YT45	CCCCGAGGATGTCAAGCATTTTCACCAG
YT104	GCTATCGCGGCCGCCAAGAAACTTGCAGAAGTACAGGACTC
YT105	GCTCACCATGCGGCCACGACCAGCTACTAAGCTCCTTTG
YT106	GTTCCTTGGGCGGCCGATGACTGGTACGTCCTAATTCGAAGAC
YT107	ACCATAGCGGCCGCCCCCGAGGATGTCAAGCATTTTC
YT149	TGTATAGAAAAGTTGACGGAGGACGATGGTAGAAACGAAC
YT150	CTACTACAGATCCCCAGAATCCGGGCGAGGACAAGG
YT151	CAACTTTTCTATACAAAGTTGATAGCTTGG
YT152	GGGGATCTGTAGTAGCTCGTG

47

Supplementary video legends

Supplementary video 1. Bidirectional motility of EEs in *A. oryzae*.

EEs were visualized by EGFP-AoRab5. Time is given in seconds and milliseconds. Scale bar, 25 µm.

Supplementary video 2. Motility of EEs in the presence of DMSO.

EEs visualized by EGFP-AoRab5 were observed under DMSO treatment. Time is given in seconds and milliseconds. Scale bar, 5 µm.

Supplementary video 3. Motility of EEs in the presence of NOC.

EEs visualized by EGFP-AoRab5 were observed under NOC treatment. Time is given in seconds and milliseconds. Scale bar, 5 µm.

Supplementary video 4. Bidirectional motility of AoHok1 in *A. oryzae*.

Although the fluorescence signal was weak, AoHok1-EGFP showed bidirectional motility. Time is given in seconds and milliseconds. Scale bar, 25 µm.

Supplementary video 5. Motility of EEs in the *Aohok1* disruptant.

EEs visualized by EGFP-AoRab5 were accumulated at the hyphal tip and showed little motility. Time is given in seconds and milliseconds. Scale bar, 25 µm.

Supplementary video 6. Bidirectional motility of POs in *A. oryzae*.

POs were visualized by EGFP-PTS1. Time is given in seconds and milliseconds. Scale bar, 25 µm.

Supplementary video 7. Motility of POs in the *Aohok1* disruptant.

POs visualized by EGFP-PTS1 accumulated at the hyphal tip and showed little motility. Time is given in seconds and milliseconds. Scale bar, 25 µm.

Supplementary Fig. 1 Togo et al

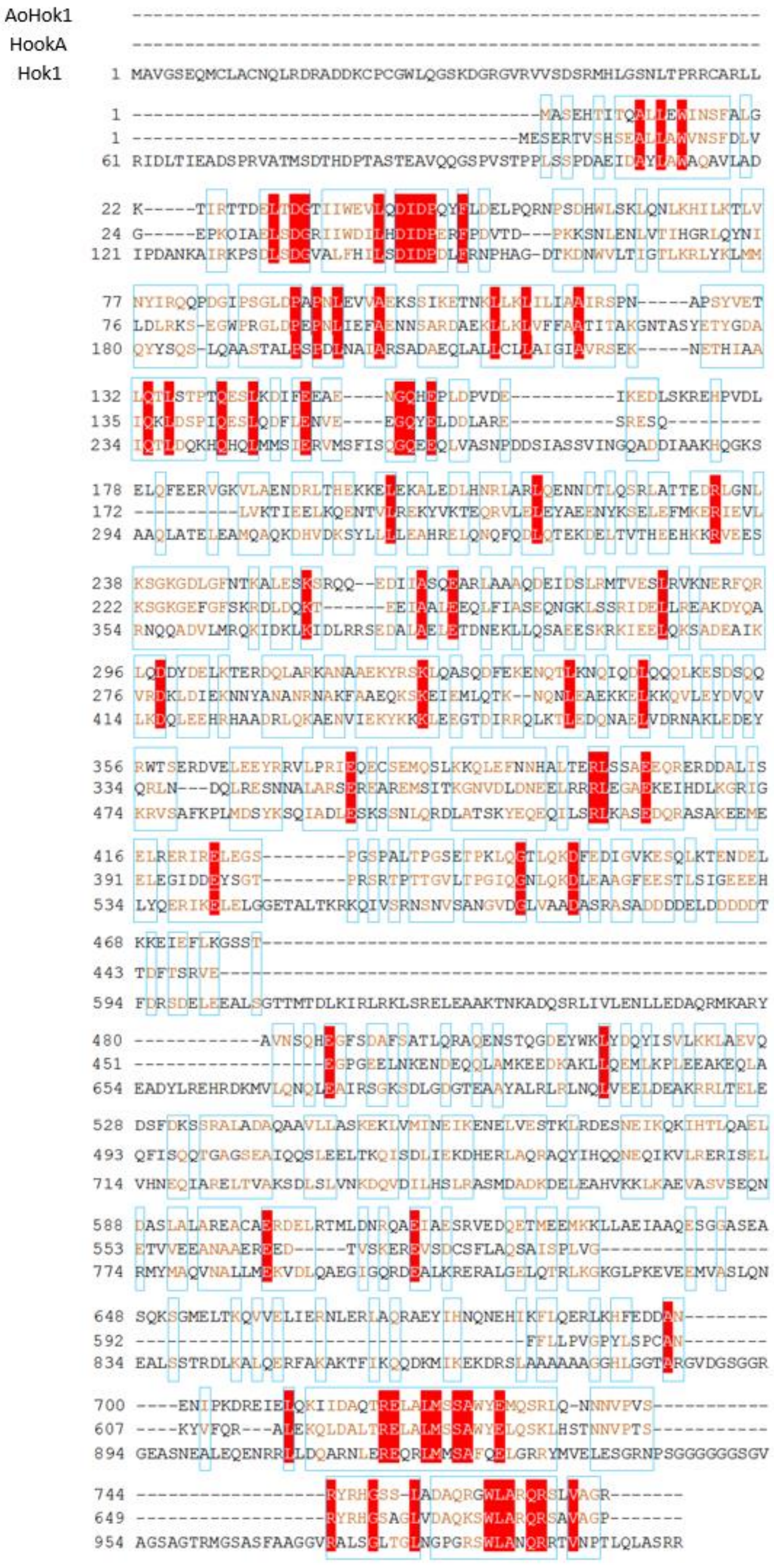

Supplementary Fig. 2 Togo et al

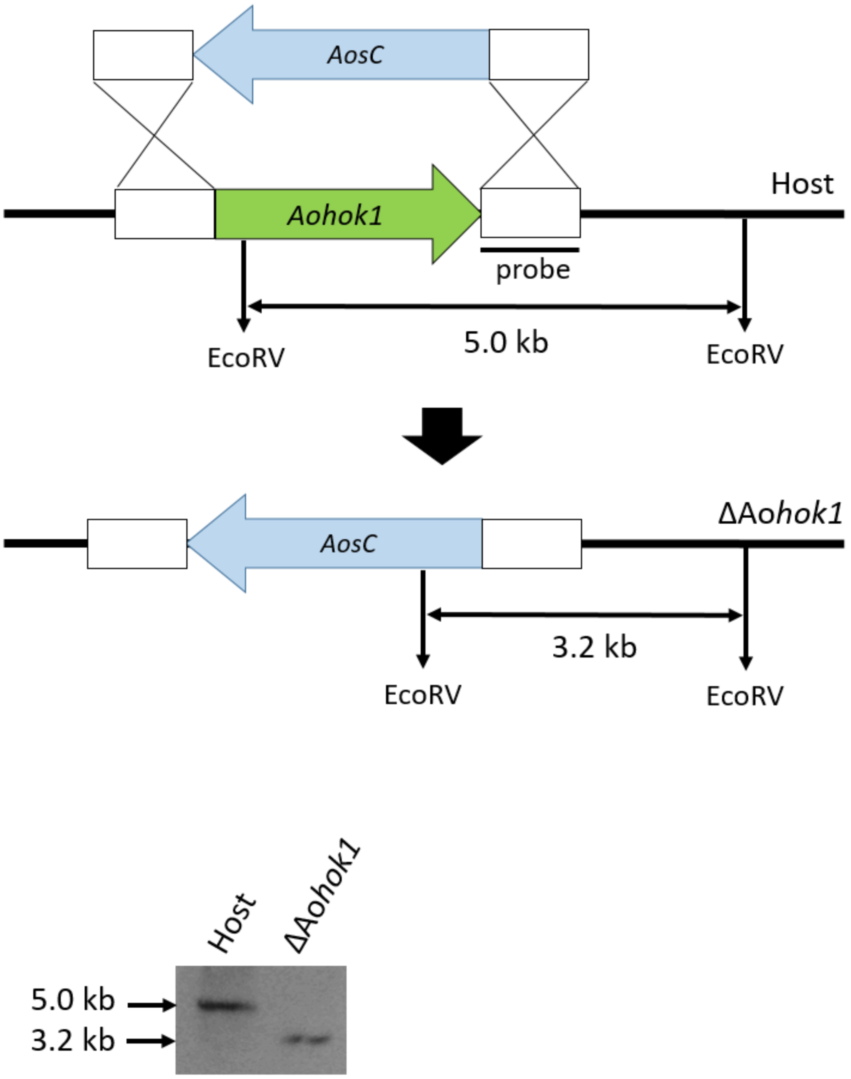

Supplementary Fig. 3 Togo et al

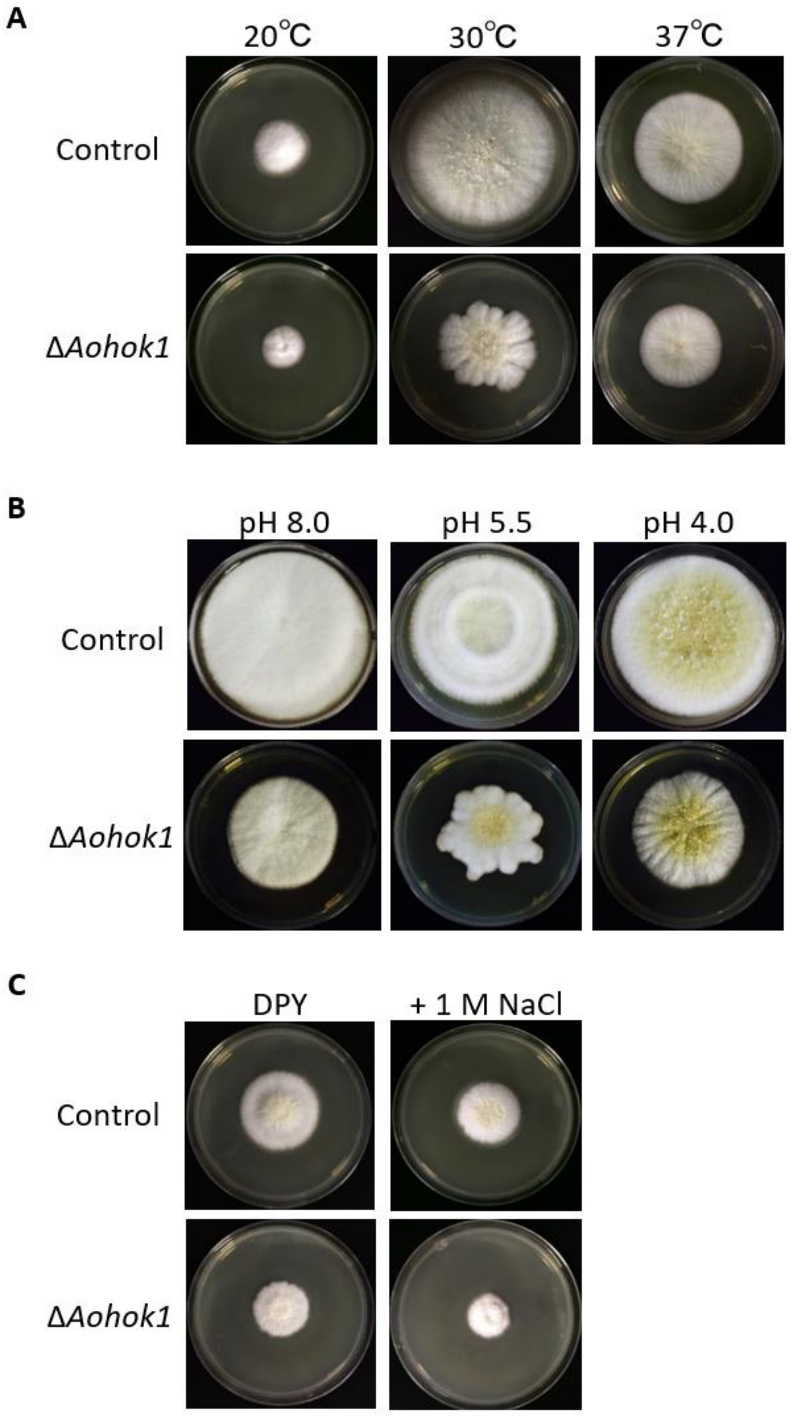

Supplementary Fig. 4 Togo et al

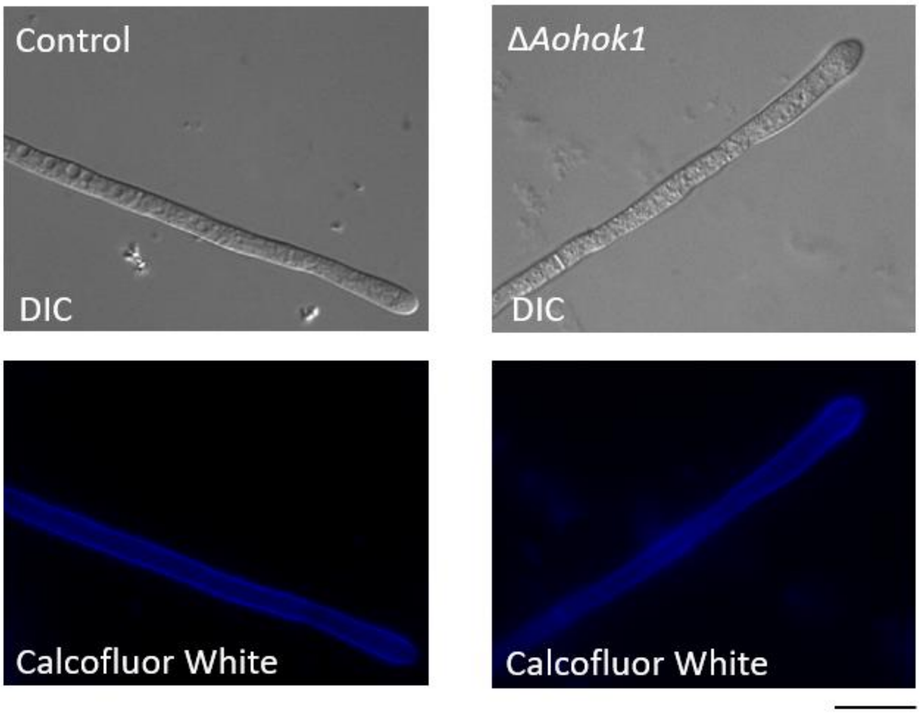

79

Supplementary Fig. 5 Togo et al

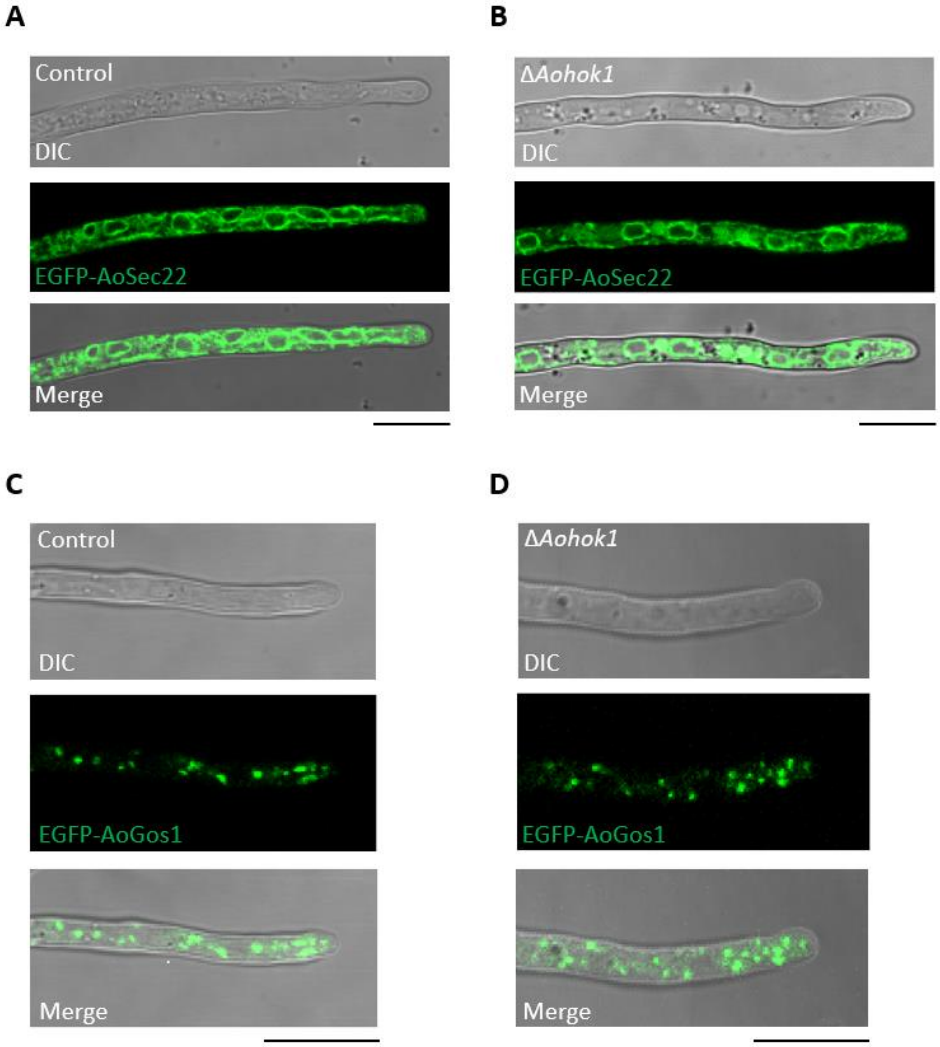

80

Supplementary Fig. 6 Togo et al

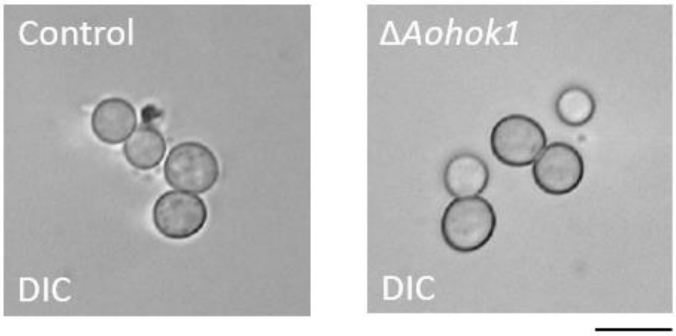

81
